# Sterility of Mammalia under Confinement

**Published:** 1881-01

**Authors:** Arthur Erwin Brown

**Affiliations:** Superintendent of Philadelphia Zoological Garden


### ART. IV.—STERILITY OF MAMMALIA UNDER CONFINEMENT.

When it is remembered that all of our domesticated animals are the descendants of progenitors which originally lived in a wild state, it will readily be conceived that the semi-domestication of wild animals in menageries seldom fails to produce constitutional changes of greater or less extent. These may be displayed in the immediate bodily form of the animal, but are more commonly confined to the deeply lying functions which serve to the perpetuation of the race. There is probably no exception to the rule that confinement lessens fertility in wild animals when they are first subjected to it. It may range in all degrees, from apparently absolute incapacity to breed, to the merely impaired vigor and irregular or incomplete development of the young, found in many species where the numerical production of offspring goes on with tolerable regularity This state of affairs is very common in zoological collections, and offers a most interesting field of observation to the comparative physiologist. Without entering, however, upon an exhaustive review of the subject, a brief statement of the facts will, perhaps, not be without interest.

It is probable that this want of productiveness may be eliminated in time, but observations to that effect are made difficult by the fact that wild animals are kept mainly for purposes of exhibition, and in such small numbers that the injurious effects of inbreeding soon enter into the question and greatly complicate the result; it is universally acknowledged, however, that domesticated races of animals are more prolific than their nearest wild congeners, although there is some evidence to show that very great changes in the conditions of life do, in some cases, lessen the fertility of animals which have long been domesticated.* Man is, as has been said, "the most highly domesticated of all

* Darwin: "Animals and Plants under Domestication." Vol. II., p. 144.

animals," and in him the generative system has reached its most perfect freedom from the influences of surroundings—under all climates, all seasons, all changes, he perpetuates his race with uniform regularity, adapting himself to conditions which would otherwise bear severely upon him in this respect. On descending, however, to the highest type of structure to be found among the lower animals, the reproductive faculties are seen to be impaired almost to the greatest degree. Few instances of monkeys breeding in confinement are to be found. Among the higher apes there has been but a limited field of observation, the few that have been kept generally being too young to afford full scope for the exercise of the sexual instinct—they all seem exceptionally delicate, however, in constitution, and there is no reason to suppose that they would be free from the conditions existing among their kindred.

During a period of twenty years, out of seventy-five species of monkeys and lemurs, kept in the London Zoological Garden, but twelve produced young; and in the Philadelphia Garden, in six years, during which time as many as sixty individuals were kept together, affording the fullest possible opportunity for intercourse, but five births took place, belonging to three species of the genus *Macacus*. In the group of American monkeys but two cases are recorded, one belonging to the genus *Cebus*, in London, and a marmoset in Paris. It is stated by Rengger and Azara that these monkeys will not even breed in their native country when kept as pets.

Among the *Cheiroptera*, several of the large fruit-eating bats have bred in Europe, but so far as may be known, no such event has occurred in the carnivorous branch of the order.

Of all the true *Carnivora*, the cats appear to be most fertile, several species—as the lion and leopard—breeding with some readiness, the tiger rarely, and the smaller species with more or less freedom. It is worthy of note that the female tiger apparently breeds more readily with the male lion than with one of her own species. Even where absolute sterility is less marked, however, a disturbance of the reproductive system is commonly shown by a tendency to abortion, by a strange perversion of instinct which, in many cases, leads the mother to devour her young, and by weak constitution and generally poor development in the offspring.

The hyænas seem to reproduce with facility, as likewise do some of the wolves, though to a less extent. Few of the foxes have ever been bred in confinement, and those which have, were for the most part hybrids with other species or with some variety of the domestic dog. The small carnivores breed with much difficulty, and in few cases, but it is among the bears that the disinclination seems to reach its maximum—the only cases of which record can be found, being confined to the polar bear and the common black bear of North America, while in England, hybrids were once produced between this species and the brown bear (*Ursus arctos*) of Europe. Of the other plantigrade carnivores, the common raccoon and crab-eating raccoon have bred a few times, and an allied species, the coati, of Central America, has produced young on several occasions, both in London and Philadelphia. Seals have hardly been kept in sufficient numbers to arrive at any sound conclusion. The common seal (*Phoca vitulina*) once produced young in the London Garden, and a cub of a species belonging to a distinct family of the order (*Zalophus gillespii*) was born in the garden at Cincinnati; but in the first of these cases, and probably also in the last, impregnation had taken place before the capture of the animal. In the fall of 1879, however, a female *Zalophus* died in Philadelphia which had been in the garden for two years and a half, and on opening the uterus an embryo of some weeks' development was found, thus showing the capacity of the species to breed in captivity. There is a noticeable want of regularity in the rut of these animals in comparison with what appears to take place in a condition of nature.

Most of the *Rodentia* seem to breed with somewhat less readiness than the members of the last group—in London, eleven out of fifty-two, and in Philadelphia, eight out of forty-one species having bred, and in almost every case with an apparent diminution in the number normally contained in a litter. Several species of the South American genus, *Dasyprocta*, are among the freest breeders, and the capromys has bred several times, producing only from one to three at a time. None of the true squirrels have ever shown any inclination to breed in confinement, though several allied species, as the woodchuck and prairie dog, do so frequently.

The elephant has, until recently, been considered as the most striking instance of complete suppression of the sexual functions, few cases of birth having taken place even under the conditions of semi-domestication in which they are kept in India. The example has been somewhat weakened, however, by the birth in March, 1880, of one of the Indian species, in the menagerie of Messrs. Cooper & Baily, at Philadelphia, and by the apparent pregnancy at the present time, of another female in their collection, by the same male.

It is among the ungulates that the nearest approach to free breeding is found. Most of the deer, antelopes and oxen breed under very considerable diversities of climate, yet even here the loss of procreative power is strongly marked. The American elk, or wapiti, is probably one of the hardiest of the family, and of its domestication in a park, in the State of Illinois, where it had free range over several hundred acres of woodland and pasture, a most competent observer* says: "The result of my experiments shows that the confinement of this deer in parks, even of considerable extent, impairs its reproductive powers. . . . . In the wild state the female is believed to breed at two, or, at most, at three years old; the young females producing one fawn at a birth, and the old ones generally twins, and three are sometimes produced at a birth. The fact that in my grounds the females never, to my knowledge, have bred before four years old, and never, I think, more than two-thirds of them in any one year, and that twins are of very rare occurrence certainly shows a sad degeneracy."

Similar results are obtained with the common, or Virginia deer. In the mule deer (*Cervus macrotis*), which breeds with some freedom, the fawns are so weakly that, hitherto, all attempts to raise them have resulted in failure. Of six bred in the Philadelphia Garden, five showed a want of assimilative power which

* Hon. J. D. Caton, "Antelope and Deer of America," p. 295.

could not be overcome, and all died early from its results; the last one born, at the time of writing is over four months old, and though not healthy, promises better than any of its predecessors which succeeded in reaching that age. In every case the mother showed a decided want of the instincts proper to the occasion, neglecting the young one after the first few days, and giving it but a scanty supply of milk. In the last case the fawn has been raised altogether by hand.

Disturbance and irregularity in the activity of the reproductive system in the deer is also indicated by the frequent occurrence, under confinement and independently of injuries, of improper development of the antlers, which are, perhaps, the best illustration, among all mammals, of a secondary sexual character; although inferences to be drawn from such malformations, are somewhat obscured by the consideration that the extent to which they occur under nature is not accurately known.

Many of the antelope and ox tribe are reasonably fertile; the giraffe has bred a number of times in Europe, and the camels and llama domesticate with ease. The hippopotamus has bred in London and Amsterdam, and several of the young have been raised. With the rhinoceroses and tapirs sterility seems to be marked—intercourse between a male and female of the latter has been frequently observed in the Philadelphia garden, but so far with no result. Of the zebras and quaggas several species have produced in Europe, but generally when crossed. Some few of the pig family also breed—the collared peccary of Central America has done so, both in Philadelphia and London, while a very closely allied species, the white-lipped peccary, is so sterile that it rarely reproduces, even when half domesticated in its own country.

Several species of *Edentata*—sloths and armadillos—have bred within a few years past in the London garden.

The marsupials appear to follow the ungulates in order of fertility, almost all herbivorous types of the order having been represented in London by breeding species, and some of them—notably the kangaroos—have done so elsewhere. The carnivorous forms are exceedingly sterile.

The common opossum, of North America, has on several occasions produced from eleven to thirteen young in the Philadelphia garden—nearly the average number contained in a litter—but they have bred but once in a season.

Of the lowest mammals (*Monotremata*), a sufficient number have not been kept to admit of any observations being made.

The table following shows the number of species which have bred, out of the total number kept in the gardens of the London and Philadelphia Zoological Societies, the only ones from which sufficient material is at hand for such a compilation to be made:

| | LONDON. | | | PHILADELPHIA. | | |
|---|---|---|---|---|---|---|
| | NO. OF SPECIES KEPT. | NO. OF SPECIES BREEDING. | PROPORTION | NO. OF SPECIES KEPT. | NO. OF SPECIES BREEDING. | PROPORTION |
| Quadrumana, . . | 75 | 12 | 1 in 6.2 | 46 | 3 | 1 in 15.3 |
| Chiroptera, . . | 1 | | | 3 | | |
| Insectivora, . . | 1 | | | | | |
| Carnivora, . . . | 77 | 24 | 3.1 | 44 | 7 | 6.1 |
| Rodentia, . . . | 52 | 11 | 4.7 | 41 | 8 | 5.2 |
| Proboscidea, . . | 2 | | | 2 | | |
| Hyraces,* . . . | 1 | 1 | 1. | | | |
| Ungulata, . . . | 94 | 46 | 2.2 | 41 | 13 | 3.1 |
| Edentata, . . . | 7 | 1 | 7. | 4 | | |
| Cetacea. . . . | 1 | | | | | |
| Marsupialia, . . | 28 | 11 | 2.5 | 19 | 4 | 4.7 |
| Monotremata, . | 1 | | | | | |
| Totals, . . . | 340 | 106 | 3.2 | 200 | 35 | 8. |

The differences between the two tables in the proportion of breeding species, may be explained by the fact that the observations in London cover a period of twenty years,† while those in Philadelphia include only six years—the time during which the garden has been in existence—the greater length of time

* (In comparing the relative fertility of different orders, the hyrax should, perhaps, be omitted, it being the sole member of its order.)

† Sclater. Proc. Zool. Society. 1868. p. 623.

and the greater number of species kept in the former, affording a much wider field for observation.

In the present state of knowledge no one cause can be assigned sufficient to account for this general range of sterility—it cannot even be definitely said whether the change of external conditions, or the inherent constitution of the animal itself, is the most important factor in the problem, as closely allied species often show a most extraordinary difference in the rate of fertility under conditions of equal change. By a system of exclusion it is possible to shut out from special prominence, each one of the greater modifications of surroundings to which wild animals are subjected after capture. If change of climate or food be held to account for the deterioration, what could be more remarkable than that the polar bear should breed, under a temperate climate, and an almost exclusively vegetable diet, as it has done in London? or that giraffes, elands and ostriches from the hot, dry plains of Africa should reproduce with some facility, as they do in various parts of the temperate zone? or if the loss of exercise attendant on confinement in cages and small enclosures, be taken as chiefly contributing to the result, what explanation can be offered of the lessened productive power displayed by many animals, as for instance, the wapiti and common deer of America, when ranging in large parks within the limits of their natural distribution? Loss of the sexual instinct is not to be regarded as an explanation, for with many animals which do not breed at all, or with extreme difficulty, constant evidences are given of a desire to gratify the feeling; nor can impaired health be a leading cause, for many of these same species are long-lived and full of vigor under confinement.

A suggestion has been made by Mr. Herbert Spencer,* to the effect that as the nitrogenized elements of food are most necessary to the formation of the embryo, they probably also play an important part in fitting the reproductive organs for the exercise of their function, and that its impairment may be directly owing to a deficiency of such elements in the food of captive animals.

* Principles of Biology. Vol. ii, p. 462.

Considering, however, the great variety of foods used in zoological collections, it is hardly probable that a deficiency in any one class of nutritive elements would be so general as must be required by this hypothesis; furthermore, it does not seem to receive the support of the facts disclosed by the foregoing tables, as in such case we should expect to find the carnivora, whose food contains the largest possible proportion of nitrogenized matter (albumen, fibrin, musculine, and the like), and to which it it possible, under all circumstances, to give their native food, breeding with markedly greater facility than the animals of any other order; whereas they do so among mammals with only moderate freedom, and among birds, the sterility of carnivorus types amounts almost to an absolute refusal to breed. Among quadrupeds, the ungulates and marsupials breed with greater, and rodents with nearly equal facility, and with the two last the principal articles of food used in menageries are bread and potatoes, both of which are specially rich in the hydro-carbons (starch, sugar, etc.), while with herbivora generally—which, on the whole, seem to furnish the freest breeders—it is probable that a change from the fresh, green food of their life under nature, to the hay, corn and oats of domestication, is attended with a gain in non-nitrogenized material, rather than the reverse.

At present, the most that can be offered by way of explanation is, that the various changes of one sort and another, suffered by the animal, all concur to produce some constitutional modification, slight in itself, but yet enough to affect in different degrees the excessively delicate operation of the generative organs, which embody in themselves germs of every vital function. How readily these may be affected, we see further in the phenomena of hybridism, when their activity is commonly impeded, and often wholly annulled, in presence even of the slight constitutional differences which can exist between closely-allied species.

Indeed, though no direct connection has yet been traced between them, such a striking similarity appears between many of the class of facts we have been observing and those relating to the crossing of species, that it is hardly anticipating too much to say that when we shall arrive at even a partial understanding of that which lies at the bottom of the one series, we shall have some light thrown upon the now unseen causes which bring about the other.

ARTHUR ERWIN BROWN,

*Superintendent of Philadelphia Zoological Garden.*

